# Serotonin receptor 5-HT7 in *Drosophila* mushroom body neurons mediates larval appetitive olfactory learning

**DOI:** 10.1038/s41598-020-77910-5

**Published:** 2020-12-04

**Authors:** Archan Ganguly, Cheng Qi, Jeevisha Bajaj, Daewoo Lee

**Affiliations:** 1 Neuroscience Program, Department of Biological Sciences, Ohio University, Athens, OH USA; 2 Department of Biomedical Genetics and Wilmot Cancer Institute, University of Rochester Medical Center, Rochester, NY USA; 3 Present Address: Department of Pharmacology and Physiology, University of Rochester Medical Center, Rochester, NY USA

**Keywords:** Learning and memory, Synaptic transmission

## Abstract

Serotonin (5-HT) and dopamine are critical neuromodulators known to regulate a range of behaviors in invertebrates and mammals, such as learning and memory. Effects of both serotonin and dopamine are mediated largely through their downstream G-protein coupled receptors through cAMP-PKA signaling. While the role of dopamine in olfactory learning in *Drosophila* is well described, the function of serotonin and its downstream receptors on *Drosophila* olfactory learning remain largely unexplored. In this study we show that the output of serotonergic neurons, possibly through points of synaptic contacts on the mushroom body (MB), is essential for training during olfactory associative learning in *Drosophila* larvae. Additionally, we demonstrate that the regulation of olfactory associative learning by serotonin is mediated by its downstream receptor (d5-HT7) in a cAMP-dependent manner. We show that d5-HT7 expression specifically in the MB, an anatomical structure essential for olfactory learning in *Drosophila,* is critical for olfactory associative learning. Importantly our work shows that spatio-temporal restriction of d5-HT7 expression to the MB is sufficient to rescue olfactory learning deficits in a d5-HT7 null larvae. In summary, our results establish a critical, and previously unknown, role of d5-HT7 in olfactory learning.

## Introduction

The biogenic amine serotonin (5-HT) plays a crucial role in learning and memory both in invertebrates and mammals^1^. Serotonin is involved in rodent learning and memory through the modulation of synaptic plasticity in the hippocampus (e.g. LTP)^2,3^. In the marine mollusk *Aplysia*, noxious stimuli are known to increase serotonergic activity in the central nervous system (CNS), which enhances gill withdrawal, a simple reflex for studying short-term learning^4^. While the role of biogenic amine dopamine in regulating learning and memory has been well studied^5,6^, the role of serotonin in *Drosophila* learning remains largely unexplored^7^. The physiological effects of biogenic amines are mediated through its G-protein coupled receptors, although the molecular identity of serotonin receptors mediating learning and memory in *Drosophila* remains to be elucidated.

The fruit fly *Drosophila melanogaster* has been a favored genetic model to study cellular and molecular mechanisms underlying learning and memory^8–10^, due to the relatively simple brain layout and well characterized neuronal architecture^11–13^. It has been established that *Drosophila* olfactory associative learning is mediated by biogenic amines including dopamine and octopamine^14–19^. In addition to dopamine and octopamine, some studies have also implicated another biogenic amine serotonin, in certain forms of learning and memory in *Drosophila* (e.g. aversive place memory)^20^, anesthesia-resistant memory^21^ and long-term memory^22^. However, the role of serotonin in *Drosophila* short-term olfactory learning remains controversial, with some studies indicating that serotonin is dispensable for olfactory learning^23^. Most of the effect of biogenic amines on behaviors including learning and memory are mediated through their downstream receptors, which are primarily GPCRs that modulate downstream cAMP-PKA signaling, a critical pathway for learning and memory^10,24^. Additionally, the cellular cAMP regulators rutabaga1 and dunce1 which are essential for short-term and long-term olfactory learning, are preferentially expressed in the *Drosophila* mushroom body (MB)^8,25,26^, a brain region critical for olfactory learning in *Drosophila* analogous to the mammalian amygdala^27^. Recent studies have also shown an increase in intracellular cAMP levels in the mushroom body with stimuli mimicking olfactory appetitive learning^28^. While the dopamine and octopamine GPCRs regulating olfactory learning are relatively well characterized^29–35^, the identity of serotonin receptors essential for *Drosophila* olfactory learning remain poorly understood.

In *Drosophila*, there are four serotonin receptors (d5-HT1A & B, d5-HT2, d5-HT7)^36^. Of these, only the d5-HT7 receptor is known to increase intracellular cAMP^37^. Another serotonin receptor 5-HT (apAC1) was identified and characterized in *Aplysia* which increases intracellular cAMP levels and is involved in learning-related heterosynaptic facilitation^38^. Several pharmacological studies have indicated that 5-HT7 receptor mediates various forms of learning and memory in mammals including hippocampus-associated spatial memory^39,40^. We thus hypothesize that the expression of 5-HT receptors, particularly those positively coupled to cAMP in the mushroom body, may be essential for learning and memory in *Drosophila*.

In this study we show that the output of serotonergic neurons through points of synaptic contacts on the mushroom body (MB) is required for olfactory associative learning in *Drosophila* larvae. Additionally, we demonstrate that the regulation of olfactory associative learning by serotonin is mediated by its downstream receptor (d5-HT7), in a cAMP-dependent manner. We show that d5-HT7 expression specifically in the mushroom body (MB) is critical for olfactory associative learning. Additionally, we also show that spatio-temporal restriction of d5-HT7 expression to the MB is sufficient to rescue olfactory learning deficits in a d5-HT7 null larvae, thus demonstrating a clear requirement for this receptor in olfactory learning.

## Results

### Serotonin is required for olfactory appetitive learning in* Drosophila* larvae

Serotonergic neurons project on to various regions of the adult *Drosophila* brain, including the mushroom body (MB)^21,22,41,42^, a structure involved in olfactory learning^8^.

**Figure 1:**
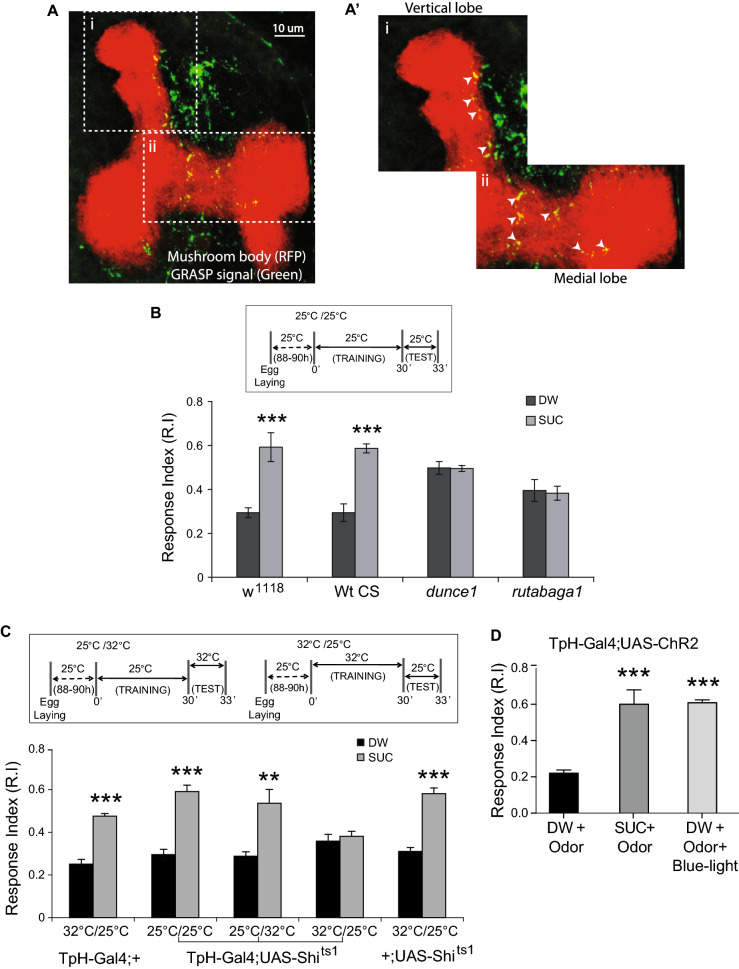
Output of serotonergic neurons is essential for olfactory appetitive learning. **(A)** Reconstituted splitGFP fluorescence (GRASP) showing regions where projections of serotonergic (5-HT) neurons (green) make synaptic contacts on to the larval mushroom body (MB) marked by the MB247-LexA signal (red). (**A′**) Enlarged view of both vertical and medial lobes from A, showing points of synaptic contact (white arrowheads) where serotonergic neurons make synaptic contacts on to the vertical lobe (**i**) and medial lobe (**ii**). Scale bar = 10 μm. (**B**) (*Top*) A schematic showing the typical larval learning regime. (*Bottom*) Wild-type strains w^1118^ (n = 7) and Canton-S (n = 6) show normal olfactory associative learning when the odor pentyl-acetate was paired with a sugar reward. The learning mutants *dunce1* (n = 6) and rutabaga1 (n = 6) fail to show normal olfactory learning. (**C**) (*Top*) A schematic of the strategy used to specifically study the role of 5-HT neurons in olfactory learning. (*Bottom*) The output of serotonergic neurons was blocked in a temperature sensitive manner by expressing the shibire protein (shi^ts1^) under the TpH-Gal4 driver. TpH-Gal4; UAS-Shi^ts1^ larvae trained at 32 °C and tested at 25 °C show impaired olfactory learning (n = 6) while larvae trained and tested at 25 °C/25 °C (n = 6) or at 25 °C/32 °C (n = 5) show normal olfactory learning. The TpH-Gal4 (n = 7) or the UAS-Shi^ts1^ (n = 7) transgenes alone displayed normal olfactory learning when trained at 32 °C and tested 25 °C respectively. (**D**) The output of the serotonergic neurons was selectively activated during the training phase by expressing channelrhodopsin 2 (ChR2) in these neurons. Blue light was used to activate the ChR2. TpH-Gal4; UAS-ChR2 larvae were exposed to odor (pentyl acetate) in the presence of blue light (BL) but no sucrose. Larvae trained in the presence of BL for 10 min (n = 6) show significantly higher RI values compared to larvae trained with odor and distilled water alone (n = 6). Student t-test: ** p < 0.01; ***p < 0.001.

While serotonergic neurons project to the mushroom body in the adult *Drosophila* brain, recent studies indicate that serotonergic neurons do not project to the larval mushroom body^23^. Most of these neuron tracing studies are based on GFP expression driven by Gal4 drivers which are not ideally suited for tracing of synaptic contacts. Additionally, the role of serotonin in *Drosophila* olfactory learning has remained controversial^20–23,41^. To conclusively define the role of serotonin in *Drosophila* larval olfactory learning, we first asked if serotonergic neurons make synaptic contacts on to the mushroom body in *Drosophila* third instar larvae. To address this, we utilized the GFP Reconstitution Across Synaptic Partners (GRASP)^43,44^ method to examine if serotonergic neurons project on to the mushroom body. We selectively labelled points of reconstituted GFP between serotonergic neurons and the larval mushroom body lobes (Kenyon cell axons) by crossing the MB247-LexA; Trh-Gal4 strain with the LexAop-rCD2.RFP; UAS-CD4-spGFP1-10, LexAop-CD4-spGFP11 strain. We detected distinct reconstituted GFP signal on both the vertical and medial lobe of the mushroom body by using a monoclonal anti-GFP antibody (Fig. 1A) while the mushroom body was labelled by RFP in a LexAop dependent manner. The reconstituted splitGFP signals are mainly observed in the upper vertical lobe (UVL) compartment and the shaft (SHA) compartment of the medial lobe (Fig. 1A). Both these regions of the mushroom body have been implicated in *Drosophila* larval learning^5,15^. Interestingly, this innervation pattern is similar to that seen for the MBIN-e2 neuron reported by Eichler et al.^11^, which is negative for neurotransmitters dopamine or octopamine. Thus, our results clearly demonstrate that points of synaptic contacts exist between serotonergic neurons and the mushroom body of *Drosophila* larvae, with innervation patterns unlike any dopaminergic or octopaminergic neuron.

Next, we wanted to determine if the output of serotonergic neurons is involved in olfactory learning in the *Drosophila* larvae. To address this we used the simple olfactory learning assay developed by Furukubo-Tokunaga et al.^14,15^ in combination with the Gal4-UAS binary expression system^45^. As a validation of the larval learning assay, we show that the two wild type strains (Canton-S and w^1118^) could successfully associate pentyl-acetate (odor) with sucrose when compared to odor and water. However, two well characterized olfactory learning mutants, *dunce1* and *rutabaga1* were unable to successfully associate odor with sucrose resulting in impaired olfactory learning (Fig. 1B). Additionally, both naïve olfactory response and gustatory response to 1 M SUC were similar to wild-type controls (Table 1). To determine the contribution of serotonin on olfactory learning, we specifically expressed the temperature sensitive mutant of dynamin (*Shibire*), in the serotonergic (5-HT) neurons of the *Drosophila* larvae using a serotonin specific Gal4 driver (TpH-Gal4) with expression pattern similar to Trh-Gal4 in *Drosophila* larvae^23^. The *shibire* mutants show a temperature dependent blockage of pre-synaptic neurotransmitter release at 32 °C but exhibit normal physiological function at 25 °C^46^. By training and testing the TpH-Gal4; UAS-Shi^ts1^ larvae at 25 °C/32 °C and at 32 °C/25 °C respectively, we specifically blocked neurotransmitter release during memory recall and the memory acquisition phases. Inhibiting the output of 5-HT neurons, during the training phase led to impaired olfactory learning (Fig. 1C). However, inhibiting the output of 5-HT neurons exclusively during the testing phase did not impair learning. In contrast, larvae with either the TpH-Gal4 or UAS-Shi^ts1^ transgenes alone displayed normal learning when trained at 32 °C and tested at 25 °C.

**Table 1:** Sensory response index. Naïve olfactory and gustatory response was carried out as described in methods. Sensory responses reported as Mean±SEM. No significant difference (*p* < 0.5) was observed between genotypes when compared using the Student’s T-test or the Mann–Whitney U test. N = 5 for all genotypes.

Genotype	Olfactory response (Pentyl-acetate) Mean ± SEM	Gustatory response (1 M SUC) Mean ± SEM
w^1118^	0.31 ± 0.043	0.40 ± 0.015
Wt (Canton-S)	0.33 ± 0.03	0.40 ± 0.008
*dnc*^*1*^	0.47 ± 0.028	0.45 ± 0.011
*rut*^*1*^	0.39 ± 0.049	0.41 ± 0.008
d5-HT7 null	0.34 ± 0.015	0.46 ± 0.024
1407-Gal4-UAS-d5-HT7-RNAi	0.34 ± 0.032	0.48 ± 0.020

To further define the role of 5-HT neurons in olfactory learning, we used optogenetics^47,48^ to selectively activate the serotonergic neurons during learning. To this end, light-sensitive channelrhodopsin 2 (ChR2) was selectively expressed in 5-HT neurons (Tph-Gal4 driver) of *Drosophila* larvae. These larvae were then exposed to blue light (BL, 470 nm) in the presence of an odor (pentyl acetate) during the training phase. No sugar reward was included during training. Larvae exposed to BL for 10 min in the presence of odor and water, but no sucrose show a significant increase in learning (Fig. 1B). However, larvae trained with odor and water in absence of blue light showed no associative learning. These results demonstrate that serotonin conveys the unconditioned stimulus (US) and its release during odor association is sufficient for appetitive learning in *Drosophila*.

### *Drosophila* 5-HT7 receptor (d5-HT7) is required for olfactory appetitive learning

Our results suggest that the output of the serotonergic neurons is essential for olfactory appetitive learning, possibly through synaptic contacts between the serotonergic neurons and the mushroom body. However, the identity of the downstream receptor which mediates the effect of serotonin on larval learning remains unknown. In vivo imaging of the adult *Drosophila* during olfactory appetitive learning has shown elevation of cAMP levels in the mushroom body^28^. Further, classical genetics studies have suggested a role for cAMP in mushroom body during olfactory learning^25,26^. There are three subtypes of serotonin receptors in *Drosophila* which are GPCRs and they either increase or decrease cAMP levels through Gαs or Gαi/o coupled activation or inhibition of the enzyme adenylate cyclase. In addition to behavioral studies, alteration in cAMP-PKA signaling can regulate synaptic strength in neurons^1^, thereby acting as molecular mechanism essential for learning.

**Figure 2:**
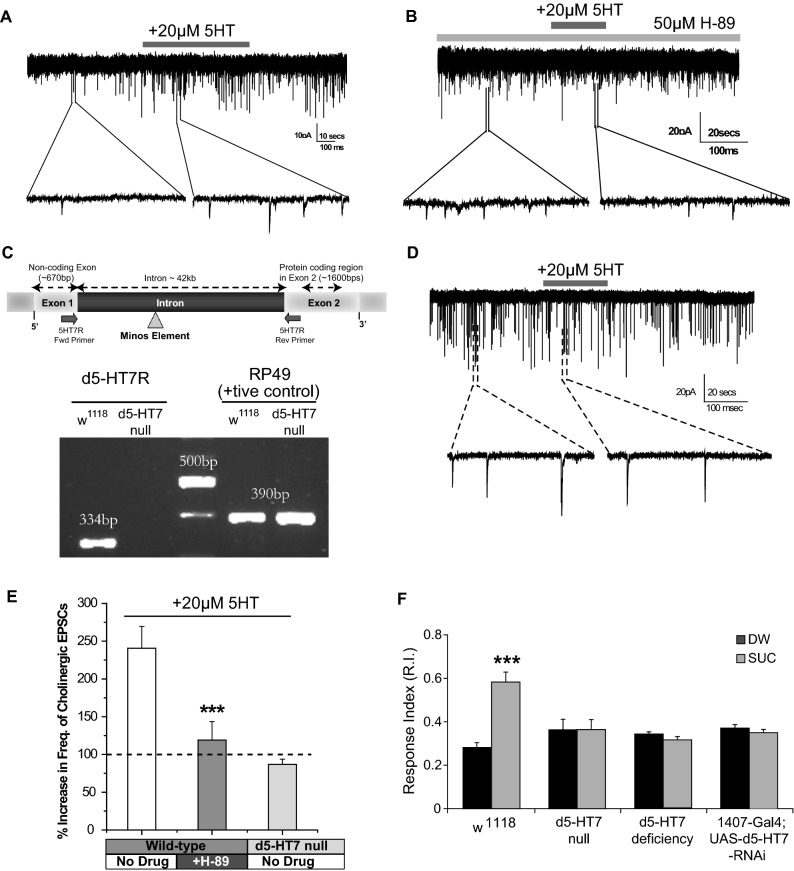
Expression of d5-HT7 receptor is essential for olfactory learning and excitatory synaptic plasticity. (**A**) Patch-clamp recording from wild-type (w^1118^) neurons in culture showing spontaneous cholinergic EPSCs (sEPSCs). Application of 20 µM 5-HT (for 30 s) shows an increase in the frequency of cholinergic EPSCs. (**B**) The application of 20 µM 5-HT (for 30 s) in the presence of a PKA inhibitor (50 µM H-89, in the bath solution) completely blocks the increase in the frequency of cholinergic EPSC. (**C**) Molecular confirmation of d5-HT7 null mutant. (*Top*) A diagrammatic representation of the d5-HT7 receptor gene in *Drosophila* showing the location of the P element insertion and the exon-exon spanning primers for the d5-HT7 receptor gene (arrows). (*Bottom*) Gel image showing the PCR products from the d5-HT7 null mutant and wild-type (w^1118^) larvae. No PCR product was detected in the d5-HT7 null strain. RP49 gene served as a control. (**D**) In contrast to wild-type neurons, neurons cultured from the d5-HT7 null mutant show no increase in the frequency of cholinergic EPSCs by 20 µM 5-HT (for 30 s). (**E**) A cumulative plot showing the effect of 20 µM 5-HT on the normalized frequency of cholinergic EPSCs on wild-type (w^1118^) neurons (n = 9), wild-type (w^1118^) neurons in the presence of 50 µM H-89 (n = 5) and on the d5-HT7 null neurons (n = 7). Student t-test: ***p < 0.001. (**F**) The d5-HT7 receptor null mutant (n = 6) and the d5-HT7 deficiency strain (n = 5) fail to show any learning after training in contrast to wild-type (w^1118^) larvae which show a significant increase in response index after being trained with sucrose and odor compared to sucrose and distilled water (n = 7). The pan-neuronal expression of UAS-d5-HT7-RNAi by the 1407-Gal4 strain leads to impaired appetitive olfactory learning (n = 5).

We thus asked if application of serotonin alters synaptic transmission in *Drosophila* using primary neuronal culture-based assays^49^. Our experiments showed that focal application of 20 µM 5-HT for 30 s on wild-type (w^1118^) neurons resulted in a significant increase in the frequency of excitatory cholinergic postsynaptic currents (EPSCs, Fig. 2A). Inhibition of PKA (a downstream target of cAMP), by the inclusion of H-89 (a chemical inhibitor of PKA) in the bath solution blocked the effect of 5-HT on the frequency of cholinergic EPSCs (Fig. 2B).

Amongst the various serotonin receptors, only the d5-HT7 receptor is positively coupled to cAMP signaling^37,50^, indicating that this receptor may be mediating the observed increase in cAMP levels upon serotonin application. To determine the role of d5-HT7 on synaptic transmission, we identified and characterized a mutant strain with a transposable element insertion in the intron for the d5-HT7 receptor (Bloomington Stock #24705). We confirmed that this mutant lacks a functional copy of the d5-HT7 transcript by PCR analysis (Fig. 2C) and henceforth refer to this strain as a d5-HT7 null. The effect of serotonin on synaptic transmission was absent in neurons from the d5-HT7 null mutant (Fig. 2D,E) with no significant difference in the baseline EPSC frequency (before serotonin application) between wild-type and d5-HT7 null mutant larvae. These results demonstrate that serotonin-induced increase in cAMP levels in neurons is primarily mediated through the d5-HT7 receptor.

To determine the impact of d5-HT7 receptor loss on olfactory learning, we carried out olfactory learning assays with the d5-HT7R null mutant and another independent d5-HT7 deficiency strain. Our experiments showed that both these strains are deficient in olfactory associative learning (Fig. 2F) compared to the wild-type strain (w^1118^). We did not observe any significant defects in response to naïve odor or the gustatory response to 1 M SUC in the d5-HT7 null mutant (Table 1). To further confirm that this deficit in learning is due to the loss of receptor expression in the larval brain, we used a pan neuronal Gal4 strain (1407-Gal4) to express d5-HT7-RNAi in the larval brain. Our results show that the down regulation of d5-HT7 expression results in impaired olfactory learning (Fig. 2F). These experiments using the receptor null, deficiency and the knockdown RNAi strains collectively indicate that the d5-HT7 receptor expression in the larval brain is essential for olfactory learning.

### d5-HT7 receptor is expressed in *Drosophila* mushroom body

**Figure 3:**
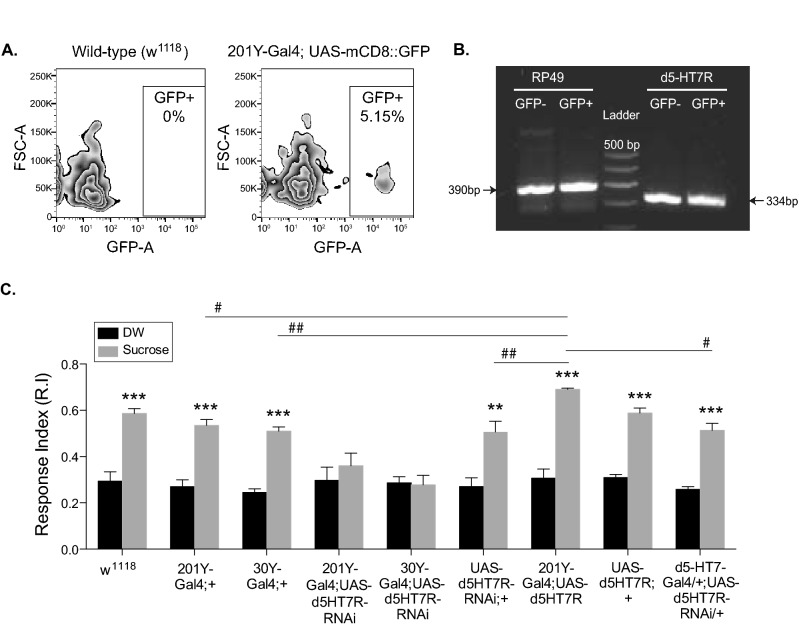
Genetic manipulation of d5-HT7 receptor expression in the larval MBs alters olfactory learning. **(A)** Fluorescence activated cell sorting (FACS) was utilized to confirm expression of d5-HT7 receptor in the larval mushroom body. FACS plots showing the gates used to isolate GFP + and GFP− neurons from larval brain of 201Y-GAL4; UAS-mCD8::GFP strain. This strain expresses GFP specifically in the MB neurons of 3rd instar larvae. Average frequency of GFP + cells obtained from three independent sorts was 5.15%. (**B**) mRNA isolated from GFP + (~ 90,000 cells) and GFP− (~ 600,000) cells sorted in (**A**) was used as a template for RT-PCR analysis using primers specific to RP49 and d5-HT7 receptor. The gel image shows that MB/GFP + neurons express the d5-HT7 receptor. (**C**) MB specific Gal4 drivers (201Y and 30Y) were crossed to UAS-d5-HT7-RNAi strain. Both the 201Y-Gal4; UAS-d5-HT7-RNAi (n = 7) and 30Y-Gal4; UAS-d5-HT7-RNAi (n = 6) strains showed impaired learning. However, the strains carrying only the 201Y-Gal4 (n = 7), 30Y-Gal4 (n = 7), UAS-d5-HT7-RNAi (n = 6) transgenes and a strain expressing UAS-d5-HT7-RNAi under 5-HT7-Gal4 (n = 5) show normal olfactory associative learning. The overexpression of d5-HT7 receptor under the MB specific Gal4 driver (201Y-Gal4; UAS-d5-HT7) resulted in higher associative learning when compared to the 201Y-Gal4/ + larvae. Student t-test: **p < 0.01; ***p < 0.001. Two-way ANOVA for comparison across genotypes: #*p* < 0.01; ##*p* < 0.001.

Our results indicate that d5-HT7 receptor expression in the brain is required for *Drosophila* larval olfactory learning. However, it is not clear if the d5-HT7 receptor is indeed expressed in the larval mushroom body. In this context, it has been shown that the d5-HT7 receptor is primarily expressed in regions outside the mushroom body^51^, and that this receptor is not required for *Drosophila* olfactory learning^52^. To conclusively determine if the d5-HT7 receptor is expressed in the MB neurons, we isolated MB neurons and probed for the presence of d5-HT7 transcripts. We used fluorescent-activated cell sorting (FACS) to isolate GFP positive MB neurons^53^ from the 201Y-Gal4; UAS-mCD8::GFP strain (Fig. 3A). This Gal4 strain expresses GFP in the MB with very little expression in the larval brain outside of the MB^17^. GFP^+^ and GFP^-^ neurons obtained from three independent sorts were pooled; the mRNA was isolated and converted to cDNA. An RT-PCR analysis for d5-HT7 receptor expression in both GFP^+^ and GFP^−^ cells show the presence of receptor transcripts in both the populations (Fig. 3B). The housekeeping gene RP49 also showed expression in both sorted cell populations. These data thus convincingly demonstrate that the d5-HT7 receptor transcript is expressed in the MB neurons of *Drosophila* larvae.

### d5-HT7 receptor in *Drosophila* MB neurons regulates olfactory learning

To establish if d5-HT7 receptor expression in the MB neurons is essential for olfactory associative learning, we identified the two 201Y-Gal4 and 30Y-Gal4 strains showing Gal4 expression specifically in *Drosophila* larvae MB neurons^14,15,17,54^. Moreover, these Gal4 strains also show GFP expression in distinct subsets of larval MB neurons^17^, making them ideal to explore the functional consequence of downregulating d5-HT7 receptor expression in the entire gamut of MB neurons.

To specifically examine the contribution of d5-HT7 receptor expression in the MB neurons on olfactory learning, we used the Gal4-UAS system^45^ to down-regulate d5-HT7 receptor (UAS-d5-HT7-RNAi) expression in the MB neurons. Both the 201Y-Gal4 and the 30Y-Gal4 strains when crossed with UAS-d5-HT7-RNAi resulted in a significant decrease in olfactory learning (Fig. 3C). However, no such defect in olfactory learning was seen in the Gal4 only, UAS-5HT7-RNAi only and in a strain in which RNAi was driven by 5-HT7 promotor (Bloomington strain# 46627). This demonstrates that the downregulation of the d5-HT7 receptor specifically in the MB neurons impairs olfactory appetitive learning.

Further, we generated a 201Y-Gal4; UAS-d5-HT7 homozygous strain, to over-express this receptor in the MB neurons. This strain shows a significantly higher response index (RI) when trained with odor and sucrose compared to similarly trained 201Y-Gal4/ + larvae (Fig. 3C). Thus, our results show that both the downregulation and over-expression of the d5-HT7 receptor in the MB neurons alters olfactory learning in *Drosophila*.

### Spatio-temporal restriction of expression in the MB neurons alone is sufficient for learning

Our results suggest a possible role of the d5-HT7 receptor expression in the MB neurons for olfactory associative learning which is contrary to studies showing that ablation of d5-HT7 receptor expression does not alter olfactory learning^52^ and that receptor expression outside the MB neurons is required for olfactory learning^51^. Thus, our results may either be due to developmental effects of receptor down-regulation or due to the non-specific effects of Gal4 expression outside of the MB neurons. To rule out both these possibilities, we carried out a series of experiments to spatio-temporally restrict receptor expression in the MB neurons.

**Figure 4:**
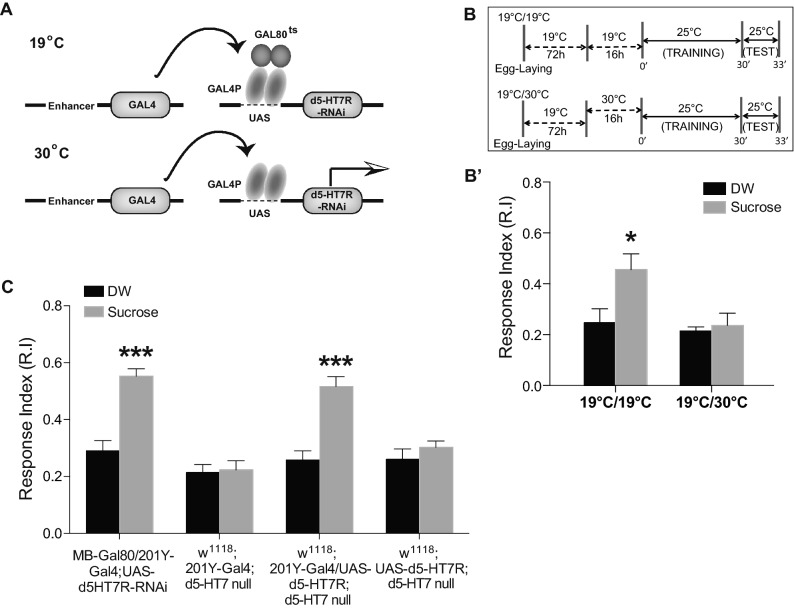
Learning defects caused by the MB specific down-regulation of d5-HT7 can be rapidly induced and are fully reversible. (**A**) Schematic representation of the Gal80^ts1^/Gal4 expression system used for spatio-temporal regulation of gene expression. At 19 °C, Gal80^ts1^ inhibits Gal4 however transfer to 30 °C denatured Gal80^ts1^ resulting in Gal4 expression. (**B**) Schematic representation of the training/testing paradigms used to assess olfactory associative learning during spatio-temporally restricted expression of d5-HT7 in the MB neurons. (**B′**) 201Y-Gal4/tub-P-Gal80^ts1^; UAS-d5-HT7-RNAi/TM6 larvae grown at 19 °C throughout development show normal learning (n = 6), but when these larvae were subjected to heat shock at 30 ºC for 16 h prior to training, it resulted in impaired learning (n = 6). (**C**) A mushroom body specific Gal80 strain (MB-Gal80) is crossed with a 201Y-Gal4; UAS-d5-HT7-RNAi homozygous strain and then subjected to learning paradigm. Gal80 inhibits Gal4 expression in the MB neurons and restores normal olfactory learning (n = 7). The expression of a wild-type copy of the d5-HT7 receptor gene in the MB neurons of the d5-HT7 receptor null larvae (201YGal4/UAS-d5-HT7; d5-HT7 null/d5-HT7 null) completely rescues olfactory associative learning (n = 7). However, the 201Y-Gal4/201Y-Gal4; d5-HT7 null/d5-HT7 null strain larvae (n = 5) and UAS-d5-HT7/UAS-d5-HT7; d5-HT7 null/d5-HT7 null strain larvae (n = 5) with the Gal4 and the UAS transgenes in d5-HT7 null mutant background, show impaired olfactory learning. Student t-test: *p < 0.05; ***p < 0.001.

The Gal80^ts1^/Gal4 expression system allows for spatio-temporal regulation of downstream gene expression^55^. We thus used this system to restrict d5-HT7-RNAi expression in the MB in a temperature sensitive manner. At 19 °C, Gal80^ts1^ inhibits Gal4 however upon transfer to 30 °C, it denatures Gal80^ts1^ resulting in Gal4 expression and UAS mediated downregulation of the d5-HT7 receptor (Fig. 4A). We crossed the 201Y-Gal4;UAS-d5-HT7-RNAi strain to the tub-P-Gal80^ts1^;TM6 strain, which expresses Gal80^ts1^ under the tubulin promoter. The corresponding F1 genotype larvae (201Y-Gal4/tub-P-Gal80^ts1^; UAS-d5-HT7-RNAi/TM6) were able to associate odor with sucrose normally when reared at 19 °C throughout. However, when the same larvae were reared at 19 °C and then moved to 30 °C for 16 h before training, they failed to associate odor with the sucrose reward (Fig. 4B′). Thus, 16 h of UAS-d5-HT7-RNAi expression in the MB neurons was sufficient to impair olfactory learning and thus rules out a major developmental defect due to impaired receptor expression in the MB neurons.

The 201Y-Gal4 strain used to down-regulate the receptor expression in the MB neurons is also known to be expressed in a small subset of neurons outside the MB^17^. This may lead to receptor expression outside the MB which may contribute to the observed defects in learning. To rule out this possibility we expressed a MB specific Gal80 (MB-Gal80)^56^ in the 201Y-Gal4; UAS-d5-HT7-RNAi strain. By blocking d5-HT7R-RNAi expression specifically in the MB neurons of the learning deficient 201Y-Gal4; UAS-d5-HT7-RNAi larvae, we show a complete rescue of the learning deficit in these larvae (Fig. 4C). The response index of these larvae was comparable to wild-type larvae. This shows that d5-HT7 receptor expression in the MB neurons alone is essential for olfactory learning.

We next sought to determine if the over-expression of the d5-HT7 receptor in a d5-HT7 mutant background could rescue the defects in olfactory learning. Expression of a wild-type copy of the d5-HT7 gene, in the MB neurons of the d5-HT7 null mutant (201Y-Gal4/UAS-d5-HT7; d5-HT7 null/d5-HT7 null), results in a complete rescue of learning and shows response index similar to wild-type larvae (Fig. 4C). In contrast, neither 201Y-Gal4; d5-HT7 null or UAS-d5-HT7; d5-HT7 null shows impaired learning like the receptor null mutants (Fig. 4C).

In conclusion, we used three independent genetic approaches to show that the expression of the d5-HT7 receptor specifically in the MB neurons is sufficient for olfactory associative learning in *Drosophila* larvae.

## Discussion

Serotonergic neurons project to various anatomical structures in the adult *Drosophila* brain including the mushroom body (MB), a structure critical for olfactory learning in the flies^21,22,42^. Although recent studies suggest that the output of serotonergic neurons is essential for certain forms of olfactory learning^20–22,41^, the evidence for serotonin’s role in olfactory appetitive learning in *Drosophila* remains rather sparse when compared to the extensive evidence for dopamine’s role in olfactory learning (reviewed in^7^).

In this study we demonstrate that the output of serotonergic neurons is essential for olfactory appetitive learning and demonstrate that its downstream receptor, the d5-HT7 (*Drosophila* 5-HT7 homolog) is critical for learning in *Drosophila* larvae. We conclusively show that the expression of this receptor in the MB neurons alone is essential for olfactory appetitive learning. Additionally, we show that d5-HT7 receptor increases intracellular cAMP levels in neurons upon the application of serotonin. In context of recent evidence suggesting that elevation of cAMP in MB neurons is associated with olfactory appetitive learning^28^, our findings strongly suggest that changes in cAMP signaling mediated by the d5-HT7 receptors forms the underlying cellular basis for olfactory appetitive learning.

A detailed anatomical characterization of MB extrinsic serotonergic neurons by GRASP reveals that serotonergic neurons form close synaptic contacts on to the lobes of the MB neurons in adult *Drosophila*^22,42^. However, in the *Drosophila* larvae, serotonergic neurons have not yet being reported to project on to the mushroom body neurons^23^. By using the GRASP technique, we convincingly demonstrate that serotonergic neurons form synaptic contacts on both the vertical and medial lobes of the mushroom body (Fig. 1A,A′). The innervation pattern of serotonergic is morphologically distinct from, and rather sparse, when compared to the innervation patterns of dopaminergic neurons in *Drosophila* larvae^5,11^ suggesting the identification of a novel circuit component in *Drosophila* larvae. Additionally, we show that the output of the serotonergic neurons is essential during the acquisition phase of olfactory appetitive learning (Fig. 1C). Further, optogenetic activation of serotonergic neurons, we show that depolarizing these neurons during training induces olfactory appetitive learning even in the absence of sucrose (Fig. 1D). Our results show that the synaptic output of the serotonergic neurons is essential for conveying the unconditioned stimulus (sucrose) during the memory acquisition phase. Our data is in contrast to the widely held view that the output of the dopaminergic neurons are exclusively responsible for conveying the unconditioned stimulus during appetitive olfactory learning^18,19,48,57^. However, our data is consistent with reports which suggest that other biogenic amines besides dopamine like octopamine, is also capable of conveying the unconditioned stimulus in larval olfactory learning^15,58^. Additionally, there are reports of the existence of neuropeptides which can convey the unconditioned sucrose reward signal during olfactory appetitive learning^59^. While the identity and neurotransmitter expression profile of the neuron projecting on to the MB neurons in our study remains unknown, our result suggest that the biogenic amines other than dopamine, like octopamine and serotonin, may provide additional layers of regulation for olfactory appetitive learning in *Drosophila* larvae. This may occur either through the same neuron co-expressing both dopamine and serotonin as was observed in the adult *Drosophila* brain^60^ or through serotonergic neurons communicating at the synaptic level with downstream dopaminergic neurons projecting on to the *Drosophila* MB lobes^22^. Whether either one of these scenarios is essential for olfactory appetitive learning in *Drosophila* larvae remains a subject for future investigation.

Elevation of cAMP levels in the MB neurons have recently been shown to be essential for both short-term^28^ and long-term olfactory learning in *Drosophila*^22^. Additionally, several recent studies show an in vivo elevation of cAMP-PKA levels in the *Drosophila* mushroom body under conditions mimicking a classic olfactory learning paradigm^22,28,61–63^. While *Drosophila* express five mammalian homologs of serotonin receptors, only one amongst them, the d5-HT7 receptor, is known to positively activate cAMP signaling^36^. This led us to examine if the activation of the d5-HT7 receptor downstream of serotonergic neurons, particularly in the MB neurons contributes to olfactory appetitive learning. By pan-neuronal suppression of this receptor in genetic mutants we demonstrate that d5-HT7 receptor expression in the larval brain is essential for olfactory appetitive learning in *Drosophila* larvae (Fig. 2F). Although the expression of the d5-HT7 receptor has been cataloged in the MB neurons of several invertebrates^36^, current evidence suggests that this receptor is not expressed in the *Drosophila* MB^23,51,64^. In fact, some studies suggest that the d5-HT7 receptor is not essential for olfactory learning in *Drosophila*^52^. We probed for receptor expression in larval MB neurons by FACS sorting to probe for receptor expression. Our results convincingly demonstrate that d5-HT7 receptor cDNA is expressed in GFP positive MB neurons (Fig. 3A,B). Additionally, we show that down-regulating d5-HT7 receptor expression specifically in the MB neurons results in impaired olfactory appetitive learning (Fig. 3C). While MB restricted expression of a serotonin receptor (d5-HT1A) and the d5-HT2A receptor have been implicated in anesthesia-resistant aversive memory^21^ and long-term memory^22^, neither of these receptors are positively coupled to cAMP signaling. Additionally, while MB limited expression of d5-HT1A receptor, which is negatively coupled cAMP signaling, is essential for sleep consolidation^65^, there is no evidence to suggest any role for the d5-HT7 receptor in *Drosophila* olfactory learning via expression in the mushroom body. Our results demonstrate a clear role for a serotonin receptor positively coupled to cAMP in *Drosophila* olfactory learning, consistent with elevation of cAMP levels in the MB neurons during olfactory appetitive learning^7,28^. Our results are also consistent with studies which suggest a role for octopamine receptors (which activate cAMP levels) in olfactory learning through expression in the MB neurons^30,31,66^. While we show strong evidence for the involvement of the d5-HT7 receptor in olfactory learning, we cannot completely rule out the possible crosstalk between different serotonin receptors in olfactory appetitive learning, possibly though expression in the MB neurons. This scenario remains a distinct possibility given the recent study highlighting the role of two distinct dopamine receptors in the *Drosophila* MB, which differentially modulate the temporal integration of odor cue with the reinforcement signal^67^.

Previous studies report that d5-HT7 receptor is expressed in several regions of the *Drosophila* brain including the central complex, large-field R neurons of the ellipsoid body, antennal lobes but is not expressed in the MB neurons^23,51^. Using FACS sorting of *Drosophila* larval MB neurons we show that the d5-HT7 receptor transcript is expressed in the MB neurons (Fig. 3A,B). While our data suggests a clear role for the d5-HT7 receptor in olfactory appetitive learning, its role has been challenged in *Drosophila* larvae^52^. In the study by Huser and colleagues, the authors demonstrate that ablation of d5-HT7 receptor expressing neurons has no effect on olfactory appetitive learning. The effect of knocking down early in development does not rule out a development contribution of the d5-HT7 receptor in olfactory learning. To rule out any development effect of d5-HT7 receptor knockdown on larval learning, we used the temperature sensitive Gal4/Gal80 system^55^ to down-regulate d5-HT7 receptor expression for a short duration before training (Fig. 4B). This allowed us to rule out any effect of receptor down-regulation on olfactory learning prior to the 3rd instar larval stage. Additionally, since the 201Y-Gal4 strain we used in our study to knockdown d5-HT7 receptor is known to express outside the MB neurons^17^, we ruled out any effect of receptor expression outside the MB neurons on larval learning. We were also able to restore normal olfactory learning by MB specific expression of d5-HT7 in receptor mutant larvae (Fig. 4C). Taken together, our results suggest that the expression of d5-HT7 receptor in the MB neurons but not outside of the MB, is essential for olfactory appetitive learning in *Drosophila* larvae. Since increase in cAMP levels have been observed by 2-Photon imaging in the *Drosophila* brain during appetitive learning^28^, we believe the d5-HT7 receptor by being positively coupled to cAMP, may play an essential role in olfactory learning. Thus, in addition to a handful of biogenic amine receptors (positively coupled to cAMP) whose expression in the MB neurons is essential for olfactory learning^30–32,66^, our data provides evidence for the d5-HT7 as a key mediator of olfactory learning.

With recent advances in 2-photon imaging, an increasing number of studies have reported that replicating conditions encountered during olfactory learning by pairing of the CS with a biogenic amine like dopamine or octopamine (known to convey the US), results in changes in cAMP-PKA levels in the MB neurons^22,28,61–63^. Additionally, enzymes regulating the cAMP-PKA signaling pathway like dunce1 and rutabaga1 has been identified as a key coincidence detectors during this process^22,62^. These results emphasize the essential role of cAMP signaling as an essential co-incidence detector in the *Drosophila* MB neurons during olfactory learning. While activation of both dopamine and octopamine neurons and pairing with CS resulted in alteration of cAMP levels in the MB neurons (reviewed in^7^), whether activation of serotonin neurons can do the same in olfactory learning is not known. By utilizing *Drosophila* primary neuronal cultures, which have been used to document changes in cAMP signaling on synaptic transmission^49,68,69^, we show that application of serotonin causes an increase in excitatory cholinergic transmission through an increase in cAMP levels. We also show that this effect of serotonin is mediated through the d5-HT7 receptor (Fig. 2A–E). Our results are consistent with studies showing an increase in cAMP levels during olfactory learning^28^. Although the effect of serotonin on in vivo cAMP levels in MB neurons during conditions mimicking olfactory learning is yet to be explored, our data strongly suggests a role for the d5-HT7 receptor in coincidence detection via elevation of cAMP in the mushroom body neurons.

## Materials and methods

### Fly strains

Wild-type flies used in all experiments were the w^1118^ (a “Cantonized” white eye stock) and the Canton-S genotype (Wt). The Tph-Gal4 line was a kind gift from Dr. J. Kim at KIAST, Korea. The UAS-5HT7R line was gifted by Dr. Julian Dow at Univ. of Glasgow^50^ and the UAS-Shi^ts1^ line was a kind gift from Dr. T Kitamoto at Univ. of Iowa^46^. MB-Gal80 strain was a gift from Dr. W. Joiner (UCSD). UAS-ChR2 was obtained from Dr. Barry Condron at Univ. of Virginia. The MB247-LexA(III) strain was a gift from Dr. Marcus Gallio (Northwestern University). All homozygous lines used in this study were made by standard genetic crosses using the w^1118^; CyO/Sco; TM2/TM6B double balancer line. For P-element mobilization of the UAS-d5-HT7 gene from the II to the III chromosome, standard genetic cross scheme was used with a strain carrying the Δ 2–3 transposase enzyme in the w^1118^ background. All the other lines used were from the Bloomington Stock Center, details of which are encapsulated below.

**Table:** 

Common name	Bloomington stock no	Complete genotype
d5-HT7 null	#24705	w^1118^; Mi{ET1}5-HT7^MB04445^ CG31008^MB04445^
d5-HT7 Deficiency	#24142	w^1118^; Df(3R)ED6346/TM6C, cu^1^ Sb^1^
UAS**-**d5-HT7-RNAi	#27273	y^1^ v^1^; P{TRiP.JF02576}attP2
*rut1*	#9404	*rutabaga*^*1*^
*dnc1*	#6020	*dunce*^*1*^
201Y-Gal4	#4440	w^1118^; P{GawB}Tab2^201Y^
c309-Gal4	#6906	w*; P{GawB}c309
1407-Gal4	#8751	w*; P{GawB}1407
30Y-Gal4	#30818	w[*];P{w[+ mW.hs] = GawB}30Y
tubP-Gal80^ts^	#7019	w[*]; P{w[+ mC] = tubP-GAL80[^ts^]}20; TM2/TM6B, Tb [1]
Trh-Gal4	#38388	w[1118]; P{w[+ mC] = Trh-GAL4.long}2
LexAop-rCD2.RFP; UAS-CD4-spGFP1-10, LexAop-CD4-spGFP11	#58755	w[*]; P{y[+ t7.7] w[+ mC] = CoinFLP-LexA::GAD.GAL4}attP40 P{w[+ mC] = lexAop-rCD2.RFP}2; P{w[+ mC] = UAS-CD4-spGFP1-10}3, P{w[+ mC] = lexAop-CD4-spGFP11}3/TM6C, Sb [1]
d5-HT7-5Gal4	#46627	w[1118]; P{y[+ t7.7] w[+ mC] = GMR70A04-GAL4}attP2

### Genetic crosses for GFP Reconstitution Across Synaptic Partners (GRASP)

To selectively label points of GFP reconstitution (indicating points of synaptic contacts) between serotonergic neurons and the larval mushroom body lobes (Kenyon cell axons), the MB247-LexA;Trh-Gal4 strain was crossed with a LexAop-rCD2.RFP; UAS-CD4-spGFP1-10, LexAop-CD4-spGFP11. Intense GFP signal was detected specifically by a monoclonal GFP antibody (mouse, ThermoFisher Scientific, CAT No. G-6539). The mushroom body were marked by RFP driven by MB247-LexA, LexAop system**.**

### *Drosophila *primary neuronal cultures and reagents

For the egg laying, flies were allowed to lay eggs for four hours on an agar plate containing yeast paste to stimulate egg laying. After four hours, the embryos were collected and immersed in a 50% bleach solution for four minutes to dechorionate and then rinsed with sterile water several times. The dechorionated embryos were then transferred to new petri dishes and moved into a laminar flow hood (ThermoForma, Forma Sci Inc, MA). A 50 μL Drummond Wiretrol capillary tube (Drummond Scientific Company, PA) was pulled on an electrode puller PP-830 (Narishige International USA Inc, NY) to produce a sharp micropipette. These sharp micropipettes were attached to a suction tube and used to harvest neuroblast cells from the midgastrula stage embryos^70^. Cells from two embryos (or only one embryo for neuronal cultures with deficiency lines carrying a balancer chromosome with GFP, eg. CyO-GFP) were plated on uncoated glass coverslips (Bellco Glass Inc, Vineland, NJ). Three such coverslips were placed in a 35 mm BD Falcon culture dish (BD Biosciences, Canada) and flooded with *Drosophila* culture medium DDM1 containing high glucose Ham’s F12/DME medium (Irvine Scientific, CA), l-glutamine (2.5 mM, Irvine Scientific, CA), HEPES (20 mM) and four supplements: 100 µM putrescine, 20 ng/ml progesterone, 100 µg/ml transferrin, and 50 µg/ml insulin (Calbiochem, CA). All supplements were purchased from Sigma (Sigma-Aldrich, MO) unless otherwise specified. The dishes were maintained in an incubator supplied with 5% carbon dioxide at 24 °C to 25 °C for up to 9 days as previously reported^49,68,71^. Half of the culture medium (1 mL) was replaced with fresh medium after 5 days in vitro. Both the wild-type and mutant neurons were cultured in a similar manner. 5-HT (Sigma-Aldrich, MO), H-89 (Sigma-Aldrich, MO), Poly-l-Lysine (Sigma-Aldrich, St. Louis MO), Pentyl-Acetate (Sigma-Aldrich, MO), Propionic Acid (Fluka) were all obtained from Sigma-Aldrich.

### Electrophysiology

Each coverslip containing *Drosophila* neuronal cultures was transferred into a recording chamber containing the following external solution (in mM): 140 NaCl, 1 CaCl2, 4 MgCl2, 3 KCl and 5 HEPES, pH 7.2. Pipettes for recordings were prepared by pulling borosilicate glass pipettes (Cat#53432-921, VWR International USA) with an electrode puller PP-830 (Narishige International USA Inc, NY). Postsynaptic currents (PSCs) were recorded with whole-cell pipettes with tip resistance of 6–8 MΩ for embryonic cultures under a 40 × objective on either a Nikon Eclipse TE200 inverted microscope (Nikon Instruments Inc., USA) or a Motic AE31 inverted microscope (Motic Corporation, Canada). All recordings were carried out with pipettes filled with internal solution containing (in mM): 120 CsOH, 120 d-gluconic acid, 0.1 CaCl_2_, 2 MgCl_2_, 20 NaCl, 1.1 EGTA and 10 HEPES, pH 7.2. An Axopatch 200B amplifier (Axon Instruments Inc., CA) was used to measure PSCs. For recording cholinergic PSCs the voltage was held at − 45 mV, the reversal potential of GABAergic currents. 20 μM 5-HT was applied to the cell for 30 s while it was still in whole cell mode using Picospritzer III (Parker Hannifin Corp., NJ). For the H-89 (PKA inhibitor) experiments, 50 μM H-89 was added to the external solution during 5-HT application. All drugs and neurotransmitters were focally delivered to the patched neuron by using a Picospritzer III (Parker Hannifin Corp., NJ).

### Larval learning assay

The larval learning assay was adapted from the one described earlier^14,15^. Briefly, 3rd instar larvae, 84–87 h after egg laying on fly food were separated from the fly food by using 15% glucose solution. Parental flies not more than 15 days old were used for egg laying. Since the density of the larvae was less than that of the fly food, the larvae floated to the top and were then transferred onto a sieve. The 500 μm sieve (Newark Wire Cloth Company) retained the larvae on top of the mesh whereas small bits of food and the glucose solution passed through. The larvae were washed several times with water to remove any traces of glucose.

For the training stage about 100 washed larvae were taken on a freshly made 2.5% agarose plate (8.5 cm in diameter) containing 1 mL of 1 M sucrose solution spread as a thin film on top. The inner surface of the lid of the plate had a filter paper disc (Whatmann filter) spotted with 10 μL of undiluted odor (Pentyl acetate or Propionic acid). The lid was then put on top of the agar plate. For control experiments, a similar agar plate was spread with a thin film of 1 mL of ddH_2_O instead of the sucrose solution. About 100 larvae were put on this control plate in the same order as described above for the training plate. The larvae were allowed to associate the odor with the sucrose and distilled water for 30 min at 24.5 °C in a uniformly lit fume hood with a white table top background.

For the testing phase of the experiment, 50–100 trained larvae were lined along the center of a fresh Petri dish containing 2.5% agarose. This dish had two filter discs, each placed on the top of a 1.5 mL Eppendorf tube cap pressed down into the agar at a distance of 0.7 cm from each edge of the plate. Each cap along with the filter disc formed the center of a 3 cm semi-circle on both sides of the center line. 2.5 μL of the odor used for training was then spotted on one of the filter discs and the lid was closed. The larvae were allowed to move towards the odor and non-odor side and the response index (R.I) were calculated after 3 min as described below:

$${\text{Response}}\,{\text{Index}}\left( {{\text{R}}.{\text{I}}} \right) = \frac{{\left( {{\text{No}}.\,{\text{of}}\,{\text{larvae}}\,{\text{in}}\,{\text{the}}\,3\,{\text{cm}}{\mkern 1mu} \,{\text{ring}}\,{\text{with}}\,{\text{odor }} - {\text{ No}}.\,{\text{of}}\,{\text{larvae}}\,{\text{in}}\,{\text{the}}\,3\,{\text{cm}}\,{\text{ring}}\,{\text{without}}\,{\text{odor}}} \right)}}{{{\text{Total}}\,{\text{number}}\,{\text{of}}\,{\text{larvae}}\,{\text{in}}\,{\text{both}}\,{\text{the}}\,{\text{rings}}}}$$

Naïve olfactory response and gustatory response to 1 M sucrose was carried out as described earlier^14^.

### Optogenetic larval learning assay

The optogenetic larval learning assays were performed using protocols similar to those described in studies from our group^72^. Briefly, larvae were exposed to blue light (BL; intensity, 1 mW/mm^2^), either during testing or prior to testing on plates containing 1 mM all-trans-retinal (Sigma-Aldrich). Light intensity was measured using a Sanwa Mobiken laser power meter. To activate channelrhodopsin2, a blue light source LED (Luxeon Rebel Color LEDs, 07040 PB000-D, wavelength 470 nm) with a power supply (GW Instek, Laboratory DC power supply Model GPS-1830D) was used. The intensity of the blue light is 25 mW.

### Immunohistochemistry

For staining the *Drosophila* larval brains, the brains were dissected out using sharp forceps in ice cold dissecting saline solution. The brains were then fixed in 4% paraformaldehyde for 30 min on ice and then washed thoroughly with PBS. The brains were then permeabilized in 10 mM PBSTX (PBS + 0.1% Triton X-100) and 5% Normal Goat Serum (Sigma-Aldrich, MO) for one hour at room temperature on a rocker. Primary antibody incubation was overnight at 4 °C. The following day, after three 30 min washes at room temperature in PBSTX, secondary antibody incubation was carried out for 2 h at room temperature (adult brains) or overnight at 4 °C (larval brains). The secondary antibody was diluted in the blocking solution. The brains were then washed thoroughly before mounting and imaging.

### Confocal imaging

Larval brain images were taken using a Zeiss Laser Scanning Microscope 510 (Carl Zeiss, Inc., USA). For the larval mushroom body images, 1.0 μm z-sections were taken at 100 × magnification and then stacked to create a maximum intensity projection image using the Zeiss LSM Zen 2008 software**.**

### Reverse transcription PCR analysis

Exon1-Exon2 boundary spanning primers were designed for the 5-HT7R gene homolog in *Drosophila melanogaster*. Primers for the RP49 housekeeping gene were used as a positive control. The *Drosophila* mRNA sequence was used in PrimerBlast (NCBI) to design the primers. Primers for the 5-HT7R homolog gene (Forward Primer: TCGTTGACCCAGTTCCCGACGA; Reverse Primer: CCAGTGCTGACCTGCTGCCC) and RP49 primers (Forward Primer: GAGAACGCAGGCGACCGTTG; Reverse primer: TGACCATCCGCCCAGCATAC) were obtained from Eurofins MWG Operon Inc. USA. These sets of primers were designed to yield a product size of 334 base pairs (5-HT7R) and 390 base pairs (RP49) with the isolated mRNA. Exon-exon spanning primers allowed elimination of products amplified from contaminating genomic DNA, as this would give a larger sized product than that expected with only mRNA. The mRNA was isolated using the RNAeasy Mini Kit (Qiagen Inc., CA) by following the protocol for animal tissue samples described in the product manual. 16–18 3rd instar larvae were isolated for each genotype and crushed in 300 μL RLT buffer using a pestle in a 1.5 mL tube. The lysate was passed through the QiaShredder (Qiagen Inc., CA) for homogenization and then an isolation protocol was followed as described in the product manual. After RNA isolation, the RNA was quantified using Nanodrop 1000 (Thermo Scientific, DE). Any contaminating genomic DNA was removed using the DNA-free kit (Ambion Inc./Applied Biosystems, TX) as per the manufacturer’s protocol. After genomic DNA removal, the RNA was re-quantified and converted to cDNA using the Omniscript RT Kit (Qiagen Inc., CA) for 1 μg of RNA in a final reaction volume of 20 μL as per the manufacturer’s protocol.

PCR was performed using a PCR kit (Clonetech Laboratories Inc., WI), for a final volume of 30 μL. 10 mM stock solution of each primer for both sets and 1 μL of mRNA were added to the reaction mixture in both cases. Annealing temperature for 5-HT7R and RP49 primers was 65 °C and 60 °C respectively. A standard 30 cycle PCR reaction was set up on a PTC 150 Mini Cycler (MJ Research/Bio-Rad Laboratories., CA) for both the primer sets.

For the mRNA isolation from FACS sorted GFP + and GFP− cells, a total of one million GFP + cells were taken by pooling together cells obtained from 3 separate sorts. The mRNA was isolated using the protocol described for animal cells in the Qiagen RNAeasy Micro Kit (Qiagen Inc., CA). The mRNA was converted to cDNA using the AffinityScript qPCR cDNA synthesis Kit (Stratagene, CA) for a final volume of 40 μL as per the manufacturer’s protocol. PCR was performed using a PCR kit (Takara Bio Inc., Japan) for a final volume of 20 μL. A 10 mM stock solution of each primer was used for both genes. The annealing temperature for 5-HT7R and RP49 primers was 65 °C and 60 °C respectively. A 40 cycle PCR reaction was set up on a PTC 150 Mini Cycler (MJ Research/Bio-Rad Laboratories., Hercules CA) for both primer sets.

### Fluorescence activated cell sorting (FACS) of mushroom body neurons

To obtain a single cell suspension for FACS sorting, 3rd instar larvae from the 201Y-Gal4; UAS-mCD8::GFP homozygous strain (84–87 hafteregg-laying) were collected. The brains were dissected and the ventral nerve cords removed for 100 such larvae for each sort experiment. About 25 w^1118^ (wild-type) larval brains were dissected simultaneously and served as the negative control for the FACS sorting. The brains were dissected and placed in ice cold dissecting saline solution before processing. The brains were washed with 0.1% PBS solution twice and spun down in 1.5 mL Eppendorf tubes. A pestle was used to homogenize the brains before treatment with 0.25% Trypsin–EDTA (Sigma-Aldrich, MO) for 4–5 min at 37 °C. Fetal Bovine Serum (Invitrogen, USA) to a final concentration of 4% was added to stop the action of Trypsin–EDTA. The suspension was passed through a 70 μm filter (Catalog# 35 2350, BD Biosciences, CA) and re-suspended in 0.1% PBS before sorting. For the sorting procedure, a pre-sort was done on BD*Aria* FACS sorter with wild-type cells to set the gates for sorting GFP + and GFP − cells. Following this, GFP +/GFP − cells were sorted from the 201Y-Gal4; UAS-mCD8::GFP strain. The sorted cells were flash frozen in liquid nitrogen and stored at − 80 °C until mRNA isolation.

### Data analysis

Individual PSCs were analyzed using the Minianalysis detection software (Synaptosoft, Decatur, GA, USA) with threshold criteria for individual events of 7.5 pA amplitude and 7 pF charge transfer for cholinergic PSCs^49^. Only events with a fast rising and slowly decaying phase were used for analysis. For each acquired trace of data, all events were detected, and the frequency was calculated every 5 s. If the PSC frequency declined continuously over 20 s before drug application, a rundown of PSCs was suspected, and the data were not analyzed further. For the frequency analysis, only PSCs (before drug application) with a stable frequency longer than 20 s were used to calculate the control average frequency. This calculated control average frequency was then normalized as 100%. The frequency of the entire recording was compared with the control average frequency, and then the percent frequency was calculated and plotted before, during and after drug application. PSCs were analyzed in the same way and plotted in Origin 7.0 (OriginLab, MA) and Graph Pad Prism (La Jolla, CA).

All summary graphs show the means ± SEMs. Statistical comparisons with Student’s t test yielded *p < 0.05; **p < 0.01, and ***p  < 0.001 and with Two-Way ANOVA for comparison between groups yielded #*p* < 0.01 and ##*p* < 0.001. All analysis was carried out using Graph Pad Prism (La Jolla, CA).

## Supplementary information

Supplementary Figures.
